# Real-world evidence for using urine osmolality as a practical tool in tolvaptan dose optimization in patients with ADPKD 

**DOI:** 10.5414/CNP104S14

**Published:** 2025-11-28

**Authors:** Andreja Marn Pernat, Kaja Hrovatin, Valentina Černetič Korelec, Andrej Škoberne

**Affiliations:** 1 Department of Nephrology, University Medical Center Ljubljana, and; 2 Faculty of Medicine, University of Ljubljana, Ljubljana, Slovenia

**Keywords:** autosomal dominant polycystic kidney disease, urine osmolality, tolvaptan, dose titration

## Abstract

Introduction: The aim of this prospective single-center study was to evaluate the role of morning urine and 24-hour urine osmolality as biomarkers for guiding individualized dosing of tolvaptan in adults with rapidly progressive autosomal dominant polycystic kidney disease (ADPKD), with the aim of minimizing treatment discontinuations and adverse events. Materials and methods: 24 patients (13 men, 11 women; aged 21 – 54 years) received tolvaptan for 7 – 50 months. Dosing was titrated based on tolerability and achievement of urine osmolality ≤ 250 mOsm/kg, measured by freezing point osmometry in both morning spot and 24-hour urine samples. Results: 16 patients (66.7%) remained on 45/15 mg, 7 (29%) required 60/30 mg, and 1 patient (4%) escalated to 90/30 mg of tolvaptan. Morning urine osmolality decreased significantly from 404 ± 231 mOsm/kg to 153 ± 61 mOsm/kg (p < 0.001), and 24-hour urine osmolality was maintained at ≤ 250 mOsm/kg in all patients. No significant differences in osmolality were observed between the patients on 45/15 mg and 60/30 mg. Mild liver enzyme elevations occurred in 9% of 504 total measurements, with 1 patient discontinuing treatment due to a 3-fold rise in transaminases. Another patient discontinued treatment due to aquaretic side effects. Conclusion: Urine osmolality is a practical and effective biomarker for guiding individualized tolvaptan titration in patients with ADPKD. This approach enabled adequate vasopressin suppression and was associated with a low discontinuation rate, supporting its use in real-world clinical practice.

## Introduction 

Autosomal dominant polycystic kidney disease (ADPKD) is the most common monogenic inherited kidney disease and the fourth leading cause of kidney failure worldwide [1]. In Slovenia, 6 – 8% of patients with end-stage kidney disease are affected by ADPKD [2]. Tolvaptan, an oral vasopressin V2 receptor antagonist, is currently the only approved disease-modifying therapy for ADPKD [1, 3, 4]. Tolvaptan inhibits the growth of cyst, reduces renal volume expansion, and diminishes the rate of renal function decline by suppressing cyclic AMP-mediated pathways [1, 3, 4]. Current guidelines recommend gradual titration to the maximum tolerated dose, based on the tolerability of aquaretic side effects and the risk of hepatotoxicity [1, 3, 4, 5]. However, tolerability often decreases at higher doses, which are associated with an increased rate of aquaretic symptoms and liver enzyme abnormalities [1, 3, 4, 5]. Conversely, in clinical trials in non-ADPKD patients with hyponatremia, heart failure, or cirrhosis, treated with lower tolvaptan doses, no significant increase in adverse liver events was observed compared to placebo [6]. Importantly, in ADPKD patients receiving tolvaptan, greater suppression of urine osmolality was associated with slower decline of renal function, supporting its use as a biomarker of treatment efficacy [7]. 

In this prospective single-center study, we present the long-term use of tolvaptan in a single-center cohort of adult patients with rapidly progressive ADPKD. The aim was to investigate the potential role of morning and 24-hour urine osmolality as a biomarker to guide individual tolvaptan dosing, with the goal of minimizing treatment discontinuations and adverse effects. 

## Materials and methods 

A prospective single-center study was conducted at the Nephrology Outpatient Clinic of the University Medical Center Ljubljana and included adult patients with rapidly progressive ADPKD who received tolvaptan between July 2020 and November 2024. All participants provided written informed consent in accordance with the study protocol, which was approved by the National Medical Ethics Committee of Slovenia (approval number: 0120-419/2025-2711-3) and conducted in accordance with the principles of the Declaration of Helsinki and the Declaration of Istanbul. Blood and urine samples were collected at baseline and 2 weeks after the start of treatment, then monthly during the first 18 months and every 3 months thereafter. The osmolality of serum and urine was measured using an osmometer with freezing point depression. The efficacy of tolvaptan was defined as the ability to maintain urine osmolality below 250 mOsm/kg (i.e., hypotonic urine relative to plasma). First morning spot urine osmolality was measured prior to the morning tolvaptan dose to assess the effect of the preceding afternoon tolvaptan dose overnight, while 24-hour urine osmolality was measured to determine the sustained effect of tolvaptan throughout the day. The same threshold of ≤ 250 mOsm/kg was used for both biomarkers to guide dose titration. All patients started treatment with 45/15 mg tolvaptan, which was titrated up to a maximum of 60/30 mg or 90/30 mg/day, depending on tolerability and osmolality values of morning and 24-hour urine samples, with the aim of reaching and maintaining the target value in both measurements. In addition, dose escalation was recommended if the morning urine osmolality equaled or exceeded the serum osmolality in 2 consecutive monthly measurements, even if the 24-hour osmolality remained ≤ 250 mOsm/kg. 

Adverse events such as polyuria, hypernatremia, hepatotoxicity, and treatment discontinuation were monitored throughout the follow-up period. Liver injury was defined as alanine and aspartate aminotransferases (ALT, AST) or glutamyl transferase values > 2× the upper limit of normal (ULN) or 3× the baseline values or total bilirubin > 2× ULN. Treatment was also discontinued if estimated glomerular filtration rate (eGFR) fell below 20 mL/min/1.73m^2^. 

The primary endpoints of the study included the reduction of morning urine osmolality and 24-hour urine osmolality below the threshold ≤ 250 mOsm/kg during tolvaptan treatment, the distribution of final tolvaptan doses and dose adjustments guided by urinary osmolality, and whether higher doses were associated with lower urine osmolality. Secondary endpoints included the occurrence of elevated liver enzymes and discontinuation of treatment due to polyuria or hepatotoxicity. 

### Statistical analysis 

Descriptive statistics were used to summarize all variables. Continuous data were expressed as mean ± SD. To evaluate treatment response, changes from baseline in morning and 24-hour urine osmolality were analyzed using a 1-sample t-test, with 0% as the reference value. 

The differences in morning and 24-hour urine osmolality between the different groups with the final tolvaptan dose were compared using the Kruskal-Wallis test due to a possible non-normal distribution and unequal group sizes. A p-value < 0.05 was considered statistically significant. Treatment adjustment was based on clinical events, particularly the occurrence of aquaretic side effects and liver enzyme elevations. All statistical analyses were performed using SPSS Statistics (IBM Corp., Armonk, NY, USA). 

## Results 

A total of 24 patients (13 men and 11 women) aged 21 – 54 years (mean age 40 ± 9 years) were treated with tolvaptan for a duration ranging from 7 and 50 months. Ten patients had preserved renal function at the start of treatment. In the remaining patients, the mean eGFR was 47.6 ± 16 mL/min/1.73m^2^ (range: 28 – 70) at the start of treatment and decreased to 42.5 ± 17 mL/min/1.73m^2^ (range: 21 – 78) during follow-up. The dosage of tolvaptan was individually adjusted: 16 patients (66.7%) remained on a final dose of 45/15 mg, 7 patients (29.2%) required up-titration to 60/30 mg, and 1 patient (4.2%) reached a maximum dose of 90/30 mg. Polyuria was observed in all patients, with a mean 24-hour urine output of 6,100 mL/day (range: 3,600 – 8,900 mL). One patient discontinued treatment due to excessive aquaresis. 

**Figure 1:**
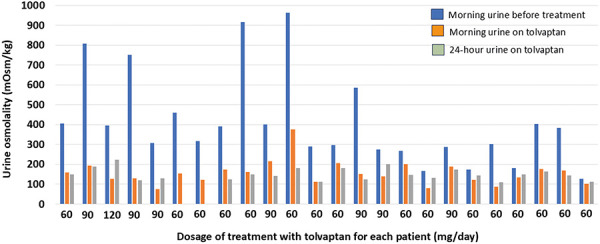
Mean osmolality of first morning urine before (blue) and during (orange) treatment with tolvaptan and 24-hour urine at the end of the study (grey) in 24 patients with ADPKD.

**Figure 2:**
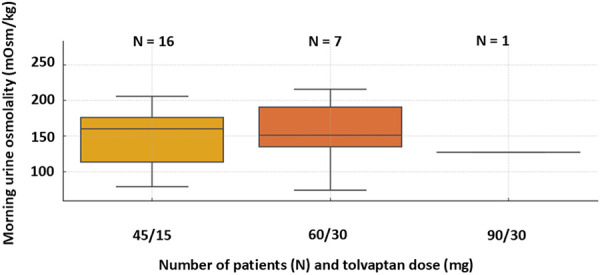
Morning urine osmolality according to dose of tolvaptan.

**Figure 3:**
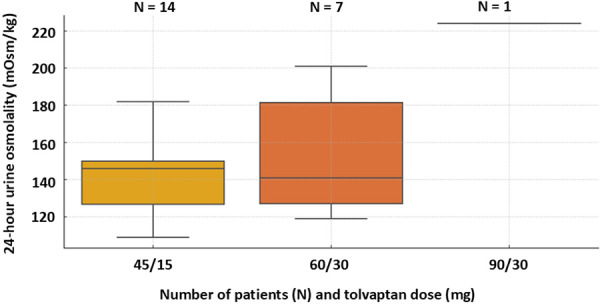
24-hour urine osmolality according to dose of tolvaptan.

Serum sodium and osmolality remained stable throughout the treatment period. Serum osmolality was 280 ± 6.5 mOsm/kg and sodium 140 ± 1.6 mmol/L at the beginning of the study, while at the end of the study it was 282 ± 7.8 mOsm/kg and 140 ± 2.3 mmol/L, respectively. As shown in Figures 1 and Figure 2, morning urine osmolality decreased significantly from a baseline value of 404 ± 231 mOsm/kg to 153 ± 61 mOsm/kg after starting treatment with tolvaptan (p < 0.001), with all but 1 patient achieving values below 250 mOsm/kg. Sustained suppression of 24-hour urine osmolality ≤ 250 mOsm/kg was achieved in all patients with a mean value of 145 ± 27 mOsm/kg (Figure 3). The comparison of osmolality across dose groups is summarized in Table 1. No statistically significant differences in morning (p = 0.823) or 24-hour (p = 0.654) urine osmolality were observed between patients receiving 45/15 mg and 60/30 mg tolvaptan. Morning urine osmolality was slightly lower with 60/30 mg compared to 45/15 mg (Table 1). The patient with the highest 24-hour urine osmolality received the highest dose of 90/30 mg (Table 1). 

Elevated liver enzyme values occurred in 9% of 504 total measurements with values up to 2× the upper limit of normal, and occurred only once in each of the 9 patients. One patient discontinued therapy due to a 3× increase in aminotransferases, which resolved after drug cessation. No hepatoxicity was observed in 7 patients (29%) who were monitored beyond 12 months. 

## Discussion 

In this prospective, single-center real-world cohort study, we aimed to demonstrate that maintaining urine osmolality ≤ 250 mOsm/kg, in both 24-hour collections and morning samples, is a possible approach to guide tolvaptan dose titration in patients with rapidly progressing ADPKD. Approximately two-thirds of patients achieved target urine osmolality with the starting dose of 45/15 mg, while 29% required escalation to 60/30 mg and 1 patient required the maximum dose of 90/30 mg. Mean morning and 24-hour urine osmolality values were within the relevant range of ≤ 250 mOsm/kg in patients on 45/15 mg and 60/30 mg, indicating adequate vasopressin V2 receptor blockade. There was no consistent trend towards lower urine osmolality at higher doses, suggesting that a fixed high-dose strategy may not be necessary for effective suppression in all patients. 

Our findings are consistent with those of Roca Oporto et al. [8] who proposed urine osmolality-guided dosing as a more appropriate strategy than targeting the maximum tolvaptan dose. In their cohort of 40 patients, 24-hour urine osmolality was used for titration, and most patients maintained osmolality ≤ 200 mOsm/kg with just 45/15 mg. Only 5% required escalation to 60/30 mg, and none required 90/60 mg of tolvaptan. This resulted in a low dropout rate (5%) and no hepatotoxicity while treatment efficacy was maintained with a > 50% reduction in eGFR slope compared to the period preceding treatment [8]. Recent commentaries have further supported this approach. Dahl and Torres [9] emphasized that lower doses of tolvaptan may often be sufficient to achieve the desired suppression of vasopressin activity and that increasing to the maximum tolerated dose may not provide additional benefit once urine osmolality is reduced below 250 – 280 mOsm/kg. Similarly, De Rosa et al. [10] highlighted the importance of urine osmolality to individualize dosing which may improve tolerability, adherence and outcomes. 

We have decided to integrate both the osmolality of 24-hour and morning urine into our titration algorithm. While 24-hour urine reflects average daily vasopressin suppression, morning spot osmolality prior to the first dose better captures the overnight effect of the afternoon tolvaptan dose, and may serve as a sensitive marker for inadequate nocturnal suppression. Therefore, we escalated therapy not only when 24-hour osmolality exceeded ≤ 250 mOsm/kg, but also when morning urine osmolality exceeded serum osmolality for 2 consecutive months, even when 24-hour osmolality remained suppressed. This may explain why 33% of patients in our cohort required tolvaptan dose escalation, compared with only 5% in the Spanish cohort of Roca Oporto et al. [8]. 

Our results contrast with real-world retrospective data from 134 ADPKD patients treated at two U.S. centers, where 27% discontinued tolvaptan within the first 2 years, primarily due to intolerable aquaresis, and approximately two-thirds required a dose reduction, leaving most patients on 45/15 mg [11]. In contrast, both our study and that of Roca Oporto et al. [8] demonstrated high treatment adherence and tolerability of aquaresis when urine osmolality-guided titration was used, with only 1 of 24 and 2 of 40 patients, respectively, discontinuing treatment due to aquaretic side effects. These findings reinforce the idea that aquaretic symptoms are manageable and compatible with daily life if unnecessary overexposure to tolvaptan is avoided. Moreover, the significant and sustained reduction in urine osmolality observed in our cohort reflects not only adequate vasopressin suppression but also strong patient commitment to treatment adherence. Despite an average 24-hour urine output of about 6 L/day, serum sodium and serum osmolality remained stable, indicating that polyuria was well tolerated and did not result in clinically significant electrolyte disturbances. This further supports the clinical benefit of avoiding unnecessary high dosing in favor of a biomarker-guided titration strategy. In addition, we observed a low incidence of hepatotoxicity. Transient elevations in liver enzymes values occurred in only 9% of the 504 measurements, and treatment discontinuation was required in a single patient due to a 3-fold increase in transaminases. These findings compare favorably with clinical trial data such as TEMPO 3:4 and REPRISE, where fixed high-dose regimens were associated with a higher incidence of hepatic adverse events [3, 4, 5]. Our findings are consistent with previous data from non-ADPKD populations, in which lower doses of tolvaptan, as used in hyponatremia, heart failure, or cirrhosis, were not associated with clinically relevant liver injury [6]. 

Our data challenge the maximum dose protocols used in early randomized controlled trials. In TEMPO 3:4, only patients (~ 80%) receiving 90/30 mg of tolvaptan consistently achieved a morning urine osmolality < 300 mOsm/kg [12]. Those who could not tolerate ≥ 60/30 mg of tolvaptan were excluded from the REPRISE study [5]. In contrast, 66.7% of our patients achieved the target of ≤ 250 mOsm/kg for both morning and 24-hour urine osmolality with 45/15 mg and a further 29% achieved it with 60/30 mg of tolvaptan. Data from TEMPO 3:4 and subsequent analyses have shown that a decrease in urine osmolality by 200 – 300 mOsm/kg, and reaching a sustained level of ≤ 200 – 300 mOsm/kg are predictive of a slower decline in eGFR [7, 8, 13]. Altogether, this supports the role of urine osmolality not only as a marker of V2 receptor inhibition and pharmacodynamic effect, but also as a predictive biomarker for long-term treatment benefit. Given the variability in renal concentrating ability among patients with ADPKD, monitoring the reduction in urine osmolality from baseline may help guide individualized tolvaptan dose titration and improve treatment tolerability without compromising efficacy. 

Our study is limited by the small sample size and single-center design and lack of a control group. In addition, our primary endpoints, urine osmolality and dose tolerability, are surrogate markers and not direct measures of disease progression. Nonetheless, our data represent a well-monitored real-world cohort and support the feasibility of urine osmolality-guided titration in clinical practice. 

## Conclusion 

Our results suggest that individualized titration of tolvaptan using morning and 24-hour urine osmolality < 250 mOsm/kg as a surrogate for vasopressin-2 receptor inhibition may be a practical, safe, and effective strategy in patients with rapidly progressive ADPKD. This approach could optimize tolerability and improve long-term adherence in routine nephrology practice. Future randomized controlled trials are warranted to compare urine osmolality-guided titration with fixed-dose escalation strategies, with a focus on long-term renal outcomes. 

## Acknowledgments 

The authors would like to thank all patients and nurses at the Nephrology Outpatient Clinic of the University Medical Center Ljubljana for their contribution to our study. 

## Authors’ contributions 

A.M.P. designed the study. A.M.P., K.H., and V.Č.K. contributed to patient recruitment and data collection. A.M.P. performed the statistical analysis and wrote the original manuscript. A.M.P. and A.Š. reviewed and edited the manuscript. All authors read and approved the final version of the manuscript. 

## Funding 

This study was funded by the tertiary research project of the University Medical Center Ljubljana (TP 20200258), Slovenia, which was approved in June 2020. 

## Conflict of interest 

The authors declare no conflict of interest. 

**Table 1.** Comparison of morning and 24-hour urine osmolality across tolvaptan doses.

Tolvaptan dose (mg)	Morning Uosm mean (mOsm/kg)	Median	24-hour Uosm mean (mOsm/kg)	Median
45/15	161	160	143	146
60/30	156	151	154	141
90/30	127	127	224	224

Uosm = urine osmolality.
